# In modern times, how important are breast cancer stage, grade and receptor subtype for survival: a population-based cohort study

**DOI:** 10.1186/s13058-021-01393-z

**Published:** 2021-02-01

**Authors:** Anna L. V. Johansson, Cassia B. Trewin, Irma Fredriksson, Kristin V. Reinertsen, Hege Russnes, Giske Ursin

**Affiliations:** 1 Cancer Registry of Norway, Oslo, Norway; 2 Department of Medical Epidemiology and Biostatistics, Karolinska Institutet, P.O.Box 281, SE-17177 Stockholm, Sweden; 3 Department of Registration, Cancer Registry of Norway, Oslo, Norway; 4 Department of Molecular Medicine and Surgery, Karolinska Institutet, Stockholm, Sweden; 5 Department of Breast and Endocrine Surgery, Karolinska University Hospital Solna, Stockholm, Sweden; 6 Department of Oncology, Oslo University Hospital, Oslo, Norway; 7 Department of Cancer Genetics, Institute for Cancer Research, Oslo University Hospital, Oslo, Norway; 8 Department of Pathology, Oslo University Hospital, Oslo, Norway; 9 Department of Nutrition, Institute of Basic Medical Sciences, University of Oslo, Oslo, Norway; 10 Department of Preventive Medicine, University of Southern California, Los Angeles, CA USA

**Keywords:** IHC subtype, ER, PR, HER2, Grade, Ki67, pTN status, TNM stage, Breast cancer, Survival/death

## Abstract

**Background:**

In breast cancer, immunohistochemistry (IHC) subtypes, together with grade and stage, are well-known independent predictors of breast cancer death. Given the immense changes in breast cancer treatment and survival over time, we used recent population-based data to test the combined influence of IHC subtypes, grade and stage on breast cancer death.

**Methods:**

We identified 24,137 women with invasive breast cancer aged 20 to 74 between 2005 and 2015 in the database of the Cancer Registry of Norway. Kaplan-Meier curves, mortality rates and adjusted hazard ratios for breast cancer death were estimated by IHC subtypes, grade, tumour size and nodal status during 13 years of follow-up.

**Results:**

Within all IHC subtypes, grade, tumour size and nodal status were independent predictors of breast cancer death. When combining all prognostic factors, the risk of death was 20- to 40-fold higher in the worst groups compared to the group with the smallest size, low grade and ER+PR+HER2− status. Among node-negative ER+HER2− tumours, larger size conferred a significantly increased breast cancer mortality. ER+PR−HER2− tumours of high grade and advanced stage showed particularly high breast cancer mortality similar to TNBC. When examining early versus late mortality, grade, size and nodal status explained most of the late (> 5 years) mortality among ER+ subtypes.

**Conclusions:**

There is a wide range of risks of dying from breast cancer, also across small breast tumours of low/intermediate grade, and among node-negative tumours. Thus, even with modern breast cancer treatment, stage, grade and molecular subtype (reflected by IHC subtypes) matter for prognosis.

**Supplementary Information:**

The online version contains supplementary material available at 10.1186/s13058-021-01393-z.

## Introduction

The prognosis of invasive breast cancer is strongly determined by tumour size (T), nodal spread (N) and distant metastases (M) at the time of diagnosis [1–4]. In addition, routine immunohistochemistry (IHC) tumour markers, i.e. estrogen receptor (ER), progesterone receptor (PR) and human epidermal growth factor receptor 2 (HER2), as well as grade and Ki67, are independent predictors of breast cancer death and have therefore together with TNM been guiding treatment decisions in the past decades [5–7].

In the early 2000s, the five intrinsic molecular subtypes of breast cancer (luminal A, luminal B, basal-like, Erb-B2/HER2-enriched, and normal breast-like) were first described, separated by gene expression analysis and with different biological properties and outcomes [8, 9]. Today, breast carcinomas can be classified into four of these types by a commercial 50-gene molecular signature [10]. This molecular classification is however recent and not yet widely used in clinical practice. Therefore, clinicians and researchers have used subtypes defined by IHC markers as proxies for the molecular subtypes [11].

Although the prognostic value of established IHC markers, TNM and grade is undisputed, they are commonly not assessed in exhaustive combinations, even in large studies [12–16]. The large registry-based epidemiological studies that have divided IHC subtypes into more detail by grade and TNM have still not fully examined the potential in the detailed stratification that is possible [17–19].

With the introduction of new treatments, it is important to continuously re-evaluate the role of classical markers for prognosis in large population-based materials to confirm and, if necessary, update the tumour/patient stratification. Such updated results will provide crucial guidance to the molecular scientists in their efforts to identify new markers for patient subgroups with insufficient prognostic characterization.

Using nationwide cancer registry data, we investigated the combined contribution of routine clinicopathologic markers (ER/PR/HER2 status, grade, tumour size and nodal status) to breast cancer-specific death up to 13 years after diagnosis. We also investigated the combined influence of these factors on early (< 5 years) and late (> 5 years) breast cancer death, in order to identify subgroups with particularly high mortality in different risk windows.

## Methods

### Study population

We identified a cohort of women diagnosed with invasive breast cancer from the Norwegian Breast Cancer Registry at the Cancer Registry of Norway (CRN) [20, 21]. The CRN has recorded new cancer cases in Norway since 1953. The registry database is considered 98.8% complete, and for breast cancer, 99.3% of cases were morphologically verified [22]. The current study included women aged 20–74 years with a primary invasive breast cancer (ICD 10 = C50) diagnosed between January 2005 and December 2015, and with no prior history of invasive carcinoma recorded in the CRN, including *n* = 24,386 women. Using ICD-O-3 morphology codes [23], we excluded tumours which were not morphologically verified (*n* = 35), not confirmed as primary (*n* = 36), or non-epithelial tumours or Paget’s disease (*n* = 154). By routine linkage to Norwegian population registries, the CRN also has information on vital status, date and cause of death and date of emigration. Women with unclear residency status at the time of cancer diagnosis were excluded (*n* = 15). After these exclusions, the cohort comprised *n* = 24,146 women.

### Histological grade and Ki67

Histological grade information was available from the ICD-O-3 code and categorized as low (I), intermediate (II) and high (III) according to the Elston-Ellis modification of the Scarff-Bloom-Richardson grading system [24]. Women with anaplastic carcinoma (*n* = 9) were excluded, leaving *n* = 24,137 women for the analysis (Additional file 1). Ki67 has been recorded routinely since 2011 (as percentage of Ki67-positive tumour cells within hot-spots) and was categorized as low (< 15.0%), intermediate (15.0–30.0%) or high (> 30.0%) according to cutoffs in the Norwegian treatment guidelines [25, 26].

### ER, PR, HER2 and IHC subtypes

Information on ER, PR and HER2 status was obtained from pathology reports for the whole study period [27]. From 2005 to January 2010, tumours were classified as ER negative (ER−) if < 10% ER expression, and from February 2010 onwards if < 1% ER expression. PR-negative (PR−) tumours were defined as < 10% PR expression throughout the study period. HER2 expression was routinely assessed with IHC and verified with in situ hybridization if the IHC results were borderline. We created six IHC subtypes: ER+PR+HER2−, ER+PR−HER2−, ER+PR+HER2+, ER+PR−HER2+, ER−PR−HER2+ (denoted HER2 positive) and ER−PR−HER2− (triple-negative breast cancer, TNBC). Women with the rarer combinations ER−PR+HER2− (*n* = 176) or ER−PR+HER2+ (*n* = 89) were set to missing in the analysis (Additional file 2). In total, *n* = 21,786 women had known IHC subtype, while *n* = 2351 women lacked information on ER, PR or HER2 status (Table 1).

**Table 1:** Clinicopathologic characteristics by IHC subtype for women with invasive breast cancer, Norway 2005–2015 age 20–74 years

IHC subtype	ER+PR+HER2−		ER+PR−HER2−		ER+PR+HER2+		ER+PR−HER2+		ER−PR−HER2+ (HER2pos)		ER−PR−HER2− (TNBC)		Missing^**a**^		Total	
	***N***	%	***N***	%	***N***	%	***N***	%	***N***	%	***N***	%	***N***	%	***N***	%
Total	13,010	100	3036	100	1424	100	834	100	1103	100	2114	100	2616	100	24,137	100.0
**Age**																
20–39	545	4.2	132	4.3	180	12.6	69	8.3	121	11.0	288	13.6	159	6.1	1494	6.2
40–49	2799	21.5	371	12.2	445	31.3	163	19.5	247	22.4	467	22.1	480	18.3	4972	20.6
50–59	4112	31.6	1015	33.4	361	25.4	305	36.6	371	33.6	639	30.2	883	33.8	7686	31.8
60–69	4489	34.5	1212	39.9	360	25.3	243	29.1	286	25.9	557	26.3	884	33.8	8031	33.3
70–74	1065	8.2	306	10.1	78	5.5	54	6.5	78	7.1	163	7.7	210	8.0	1954	8.1
**Year**																
2005–2008	3865	29.7	987	32.5	416	29.2	224	26.9	365	33.1	736	34.8	1508	57.6	8101	33.6
2009–2012	4708	36.2	1035	34.1	578	40.6	316	37.9	402	36.4	728	34.4	890	34.0	8657	35.9
2013–2015	4437	34.1	1014	33.4	430	30.2	294	35.3	336	30.5	650	30.7	218	8.3	7379	30.6
**Grade**																
I: low	3608	29.3	614	21.7	79	5.9	36	4.8	15	1.5	30	1.5	494	23.2	4876	21.9
II: intermediate	6849	55.6	1459	51.6	666	50.2	331	43.8	244	24.9	340	17.5	968	45.4	10,857	48.7
III: high	1860	15.1	752	26.6	583	43.9	388	51.4	719	73.5	1571	80.9	671	31.5	6544	29.4
Missing	693		211		96		79		125		173		483		1860	
**Ki67**^**b**^																
0–14.9%	2173	37.2	388	30.5	75	14.3	22	6.4	15	4.3	37	4.7	94	30.8	2804	29.7
15.0–30.0%	2138	36.6	384	30.2	128	24.3	106	30.9	68	19.7	64	8.1	93	30.5	2981	31.6
30.1–100%	1531	26.2	501	39.4	323	61.4	215	62.7	263	76.0	692	87.3	118	38.7	3643	38.6
Missing 2011–2015	1110		293		207		142		186		246		299		2483	
Missing 2005–2010															12,226	
**Tumour size**																
pT1, 0–20 mm	8407	72.2	1735	66.9	718	62.3	372	58.5	434	53.5	929	54.6	1415	69.9	14,010	68.2
pT2, 21–50 mm	2932	25.2	778	30.0	400	34.7	242	38.1	329	40.6	725	42.6	538	26.6	5944	28.9
pT3, > 50 mm	186	1.6	55	2.1	19	1.6	12	1.9	22	2.7	23	1.4	29	1.4	346	1.7
pT4: spread, any size	115	1.0	24	0.9	16	1.4	10	1.6	26	3.2	23	1.4	43	2.1	257	1.3
Missing	1370		444		271		198		292		414		591		3580	
**Nodal status**																
pN0, 0 nodes+	8141	67.4	1816	65.2	716	55.3	376	50.9	441	45.3	1196	63.1	1503	64.3	14,189	64.2
pN1, 1–3 nodes+	3333	27.6	819	29.4	479	37.0	282	38.2	424	43.5	599	31.6	745	31.9	6681	30.2
pN2, 4–9 nodes+	413	3.4	90	3.2	73	5.6	55	7.4	67	6.9	62	3.3	60	2.6	820	3.7
pN3, 10+ nodes+	187	1.5	60	2.2	27	2.1	26	3.5	42	4.3	39	2.1	28	1.2	409	1.9
Missing	936		251		129		95		129		218		280		2038	
**TNM stage**																
I	6619	53.0	1406	48.5	519	38.2	266	33.8	296	28.4	730	36.8	1236	50.0	11,072	48.1
II	4519	36.2	1117	38.6	589	43.3	324	41.2	465	44.6	953	48.1	887	35.9	8854	38.5
III	1056	8.5	285	9.8	185	13.6	144	18.3	225	21.6	234	11.8	225	9.1	2354	10.2
IV	287	2.3	88	3.0	66	4.9	53	6.7	56	5.4	65	3.3	123	5.0	738	3.2
Missing	529		140		65		47		61		132		145		1119	

^a^Missing on any of ER/PR/HER2 (*n* = 2351), ER**−**PR+HER2**−** (*n* = 176), or ER**−**PR+HER2+ (*n* = 89)

^b^Ki67 only available years 2011–2015

### Tumour size, nodal status and TNM stage

Pathologic T and N status was coded according to AJCC 4th edition for 2005–2008 and AJCC 6th edition for 2008–2015 [2] and categorized as pT1 (≤ 20 mm), pT2 (21–50 mm), pT3 (> 50 mm), pT4, pN0 (0 positive lymph nodes), pN1 (1–3 positive nodes), pN2 (4–9 positive nodes), pN3 (≥ 10 positive nodes) and pN+ (≥ 1 positive node), and combined as pT1pN0, pT2pN0, pT1-2pN+ and pT3-4pN0/+ according to Norwegian treatment guidelines. Patients receiving neoadjuvant treatment were missing pTN status. Pathologic TNM stage was categorized into I, IIA, IIB, IIIA, IIIB or IV [28]. This was combined with a SEER summary stage variable based on clinical data when pTNM missing into a TNM stage variable (I, II, III and IV) [2].

### Treatment

Information on the type of surgery (mastectomy, breast conserving surgery or no surgery) was available for the full study period. Information on adjuvant treatment (chemotherapy (CT), radiotherapy (RT), endocrine therapy (ET)) was only available for 57% of the women over the study period. For RT, the recorded treatment corresponded to “given” treatment. For CT and ET, the recorded treatment corresponded to “planned” treatment.

### Statistical methods

Time to breast cancer death was defined from date of breast cancer diagnosis until date of breast cancer death or censoring by death from other causes, emigration or end of follow-up in December 2017, whichever came first. The maximum follow-up was 13 years. Breast cancer-specific survival was estimated with the Kaplan-Meier method, compared with logrank tests, and age-standardized according to the internal age distribution in the sample. Breast cancer mortality rates were analysed with flexible parametric survival models (FPM) [29, 30] estimating hazard ratios (HRs) with 95% confidence intervals (CI) for combinations of IHC subtype, grade and pTN status. We estimated separate HRs during 0–5 and 5–13 years of follow-up. Models were adjusted for age and year of diagnosis and type of surgery. We did not adjust for Ki67 since it was only available from 2011. In a sensitivity analysis, we adjusted for adjuvant therapy. The baseline hazard of the FPM was estimated with a spline using 5 degrees of freedom. Time-varying effects (non-proportional hazards) were estimated with 3 degrees of freedom, and yielded smooth hazard rates shown graphically. Likelihood ratio tests assessed the interaction between IHC subtype and variables. Only women with complete information on all covariates in the adjusted models were included in the regression analyses. Frequencies of IHC subtypes by clinicopathologic characteristics were compared by Pearson chi-square tests. All tests were 2-sided and the significance level was 5%. Analyses were performed in Stata version 15.1 [31].

## Results

Among 21,786 women with known ER/PR/HER2 status, 84% were ER positive (ER+PR+HER2− 60%, ER+PR−HER2− 14%, ER+PR+HER2+ 7%, ER+PR−HER2+ 4%) and 16% ER negative (HER2 positive 5%, TNBC 10%, ER−PR+HER2− 1% and ER−PR+HER2+ 0.4%) (Additional file 2). The median age at diagnosis was 57 years (Q1–Q3 = 49–64) and the median follow-up time for breast cancer death was 6.3 years (Q1–Q3 = 3.8–9.3, range 0.0–13.0, mean 6.6).

ER+HER2− subtypes were mainly of low/intermediate grade with low/intermediate Ki67 expression, while ER− subtypes were of high grade with high Ki67 expression, with ER+HER2+ subtypes in between (Table 1). Grade and Ki67 level were highly correlated, and Ki67 level did not discriminate between grade II tumours (Additional file 3). The differences in Ki67 level across IHC subtypes were however mostly explained by grade, with the exception for ER+HER2− subtypes of intermediate grade where Ki67 did not provide discriminatory information (Additional files 4 and 5). Among ER+ tumours, PR− status conferred consistently worse grade and Ki67 compared to PR+ status (Additional file 6).

Among ER+HER2− subtypes, more than 2/3 of tumours were small (≤ 20 mm) and had no nodal spread (Table 1). Lymph node involvement was most common among ER+HER2+ and HER2-positive subtypes. ER+HER2+ and HER2-positive subtypes had the highest proportions of small tumours with nodal spread (pT1-2pN+) (range 37 to 42%), but the differences in pTN status across IHC subtypes were largely explained by grade (Additional file 7). TNM stage was most advanced among ER+HER2+ and HER2-positive subtypes overall (Table 1) and within all levels of grade (Additional file 8). PR− status conferred consistently more advanced pTN status and TNM stage compared to PR+ (Additional file 6).

### BC death by IHC subtype, grade and pTN status

To assess the independent contribution of each factor to breast cancer death, we stratified by all three variables IHC subtype, grade and pTN status in models adjusted for age, year and surgery type (Table 2). Among ER+ subtypes, an increasing grade was associated with increased mortality in all subtypes and levels of pTN status. Larger tumour size and positive nodal status were consistently associated with increased mortality in all ER+ subtypes and levels of grade, and larger size was associated with increased mortality also among node-negative tumours (*p* value for three-way interaction 0.4333). Among small tumours (≤ 20 mm) with no nodal spread, ER+PR−HER2− subtype of grade III was associated with a particularly high mortality (HR = 8.5, 4.0–18.2) and of similar magnitude to TNBC grade III tumours (HR = 9.2, 5.1–16.5). Women with larger tumours and any nodal spread (pT3-4pN0/+) had the highest mortality although numbers were low for this group. Among ER− subtypes, high-grade tumours were associated with higher mortality than intermediate-grade tumours for pT1pN0 tumours, while for other pTN status the mortality rates were similarly elevated for intermediate- and high-grade tumours.

**Table 2:** Adjusted hazard ratios for breast cancer death by IHC subtypes, grade and pTN

IHC subtype	Grade	Patients (***N***)/deaths (***n***)				pT1pN0	pT2pN0	pT1-2pN+	pT3-4pN0/+
		pT1pN0	pT2pN0	pT1-2pN+	pT3-4pN0/+				
		***N***/***n***	***N***/***n***	***N***/***n***	***N***/***n***	HR [95% CI]	HR [95% CI]	HR [95% CI]	HR [95% CI]
ER+PR+HER2−	I	2498/17	225/7	592/22	30/2	**1.0 [ref]**	3.6 [1.5, 8.6]	4.2 [2.2, 7.9]	6.3 [1.5, 27.5]
	II	3175/36	805/23	1892/117	149/25	1.6 [0.9, 2.8]	3.6 [1.9, 6.7]	7.3 [4.4, 12.2]	15.5 [8.3, 28.9]
	III	556/25	332/24	641/83	51/16	6.4 [3.4, 11.8]	10.5 [5.6, 19.7]	16.7 [9.9, 28.2]	39.8 [20.0, 79.6]
ER+PR−HER2−	I	445/2	30/1	80/5	8/0	0.7 [0.2, 2.9]	4.3 [0.6, 32.5]	7.9 [2.9, 21.5]	N/A
	II	650/16	173/10	377/50	37/8	3.3 [1.7, 6.6]	7.5 [3.4, 16.4]	14.3 [8.2, 24.9]	24.8 [10.6, 57.9]
	III	214/11	158/16	237/46	15/3	8.5 [4.0, 18.2]	16.2 [8.2, 32.2]	24.5 [14.0, 42.9]	22.3 [6.5, 76.4]
ER+PR*+*HER2+/ER+PR−HER2+^a^	II	376/6	114/5	266/21	23/2	2.1 [0.8, 5.3]	6.0 [2.2, 16.2]	8.5 [4.4, 16.1]	7.6 [1.7, 32.8]
	III	286/12	149/12	344/26	14/3	5.6 [2.7, 11.7]	11.1 [5.3, 23.3]	9.3 [5.0, 17.2]	21.3 [6.2, 73.1]
HER2pos	II	82/1	21/1	63/7	5/2	1.3 [0.2,9.4]	7.2 [1.0, 53.8]	12.9 [5.3, 31.2]	44.5 [10.2,193.5]
	III	168/10	110/8	247/28	30/8	6.7 [3.1, 14.6]	9.2 [3.9, 21.3]	12.8 [7.0, 23.4]	27.6 [11.9, 64.4]
TNBC	II	150/6	45/7	59/12	6/3	4.8 [1.9, 12.1]	17.8 [7.4, 43.1]	22.3 [10.6, 46.8]	86.9 [25.4, 297.5]
	III	488/34	368/40	393/80	27/8	9.2 [5.1, 16.5]	14.4 [8.2, 25.5]	25.8 [15.2, 43.7]	28.5 [12.2, 66.5]

Hazard ratios adjusted for age and year of diagnosis, subtype × grade × pTN status interaction, surgery type and follow-up. Restricted to M0. *N* = 17,204. Test of interaction: 3-way interaction IHC subtype × grade × pTN status vs. IHC subtype × grade + pTN status, *p* = 0.4333. Test of interaction: 2-way interaction IHC subtype × grade + pTN status vs. IHC subtype + grade + pTN status, *p* = 0.0022

*HER2pos* ER**−**PR**−**HER2+, *TNBC* ER**−**PR**−**HER2**−**

^a^ER+PR+HER2+ and ER+PR**−**HER2+ combined into one category

When combining the effect of IHC subtype, grade and pTN status, there were some subgroups with particularly high mortality (Table 2). Those were ER+PR+HER2− grade III pT3-4pN0/+ (HR = 39.8, 20.0–79.6), ER+PR−HER2− grade III pT1-2pN+ (HR = 24.5, 14.0–42.9) and TNBC grade II and III pT1-2pN+ (HR = 22.3, 10.6–46.8; HR = 25.8, 15.2–43.7, respectively). These groups included a sufficient number of events (> 10 deaths) for statistical inference, but other smaller groups are also presented for completeness.

### BC death by time-since-diagnosis

**Fig. 1:**
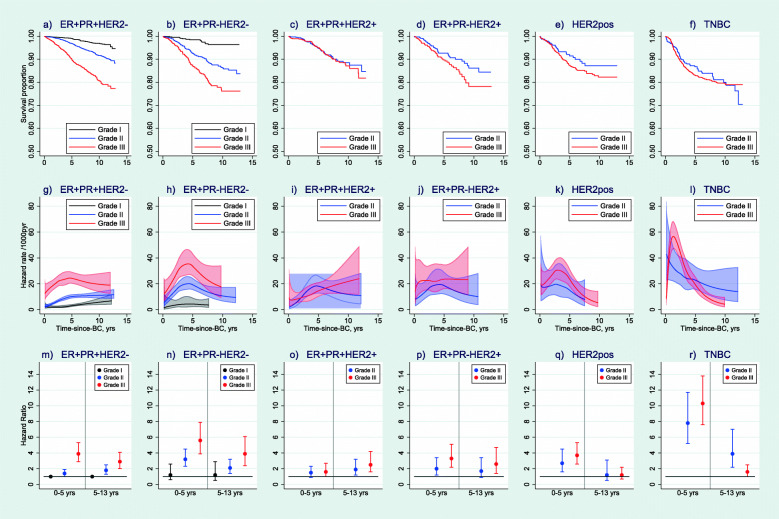
Breast cancer-specific survival proportions, hazard rates and adjusted hazard ratios by IHC subtype and grade. Legend: Survival proportions (panels **a**–**f**), hazard rates (panels **g**–**l**) and adjusted hazard ratios (panels **m**–**r**) including *n* = 19,220 women with known information on ER, PR, HER2, grade, TNM stage and surgery type. Numbers at risk at start: ER+PR+HER2− *n* = 3497 (grade I), *n* = 6605 (grade II), *n* = 1789 (grade III); ER+PR−HER2− *n* = 597 (grade I), *n* = 1399 (grade II), *n* = 717 (grade III); ER+PR+HER2+ *n* = 637 (grade II), *n* = 564 (grade III); ER+PR−HER2+ *n* = 318 (grade II), *n* = 369 (grade III); HER2pos *n* = 235 (grade II), *n* = 688 (grade III); TNBC *n* = 320 (grade II), *n* = 1485 (grade III). Logrank tests of survival differences by grade: ER+PR+HER2− *p* < 0.001, ER+PR−HER2− *p* < 0.001, ER+PR+HER2+ *p* = 0.7013, ER+PR−HER2+ *p* = 0.0836, HER2pos *p* = 0.1466, TNBC *p* = 0.6230. Hazard rate curves only plotted until last event in each group. Hazard ratios adjusted for age and year at diagnosis, subtype × grade interaction, TNM stage, surgery type and follow-up. *N* = 19,220. Estimates of HRs are given in Additional file 9

In a second step, we assessed breast cancer death by subtype and grade at early (0–5 years) and late (5–13 years) follow-up. Among ER+HER2− subtypes, survival was markedly poorer for grade III compared to grade II tumours (Fig. 1a, b), and the mortality rate remained elevated up to 13 years after diagnosis (Fig. 1g, h). Adjusted hazard ratios confirmed the association (Fig. 1m, n). Compared to ER+PR+HER2− tumours of grade I, tumours of grade II or III were associated with increased mortality both early and late, with strongest associations for grade III (early: HR = 3.9 (95% CI 2.9–5.3), late: 2.9 (2.0–4.1), Fig. 1m, Additional File 9). Stronger associations with grade were observed for ER+PR−HER2− subtype (Fig. 1b, h, n), and weaker associations for ER+PR+HER2+ and ER+PR−HER2+ subtypes (Fig. 1c, d, i, j, o, p). HER2-positive subtype of grade II or III was associated with increased early but not with late mortality (Fig. 1e, k, q). The highest early mortality was observed for the TNBC subtype of grade II or III which had eightfold and tenfold mortality rate, respectively, compared to the reference group (Fig. 1f, l, r). In all these comparisons, it is important to recall that the reference group (women with ER+PR+HER2− grade I tumours (Fig. 1g)) had an increasing mortality over follow-up (HR = 1.9, 1.4–2.7 comparing mortality at 10 vs. 1 year after diagnosis) and that ER+ high-grade tumours accounted for the largest numbers of deaths.

**Fig. 2:**
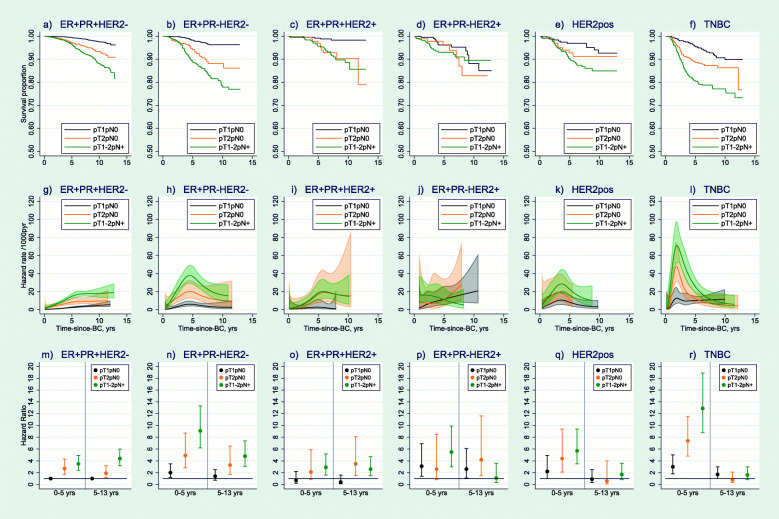
Breast cancer-specific survival proportions, hazard rates and adjusted hazard ratios by IHC subtype and pTN. Legend: Survival proportions (panels **a**–**f**), hazard rates (panels **g**–**l**) and adjusted hazard ratios (panels **m**–**r**) including *n* = 16,809 women with known information on ER, PR, HER2, grade, TNM stage and surgery type, and restricted to T1-2, N any, M0. Numbers at risk at start: ER+PR+HER2− *n* = 6229 (pT1pN0), *n* = 1362 (pT2pN0), *n* = 3125 (pT1-2pN+); ER+PR−HER2− *n* = 1309 (pT1pN0), *n* = 361 (pT2pN0), *n* = 694 (pT1-2pN+); ER+PR+HER2+ *n* = 437 (pT1pN0), *n* = 171 (pT2pN0), *n* = 380 (pT1-2pN+); ER+PR−HER2+ *n* = 225 (pT1pN0), *n* = 92 (pT2pN0), *n* = 230 (pT1-2pN+); HER2pos *n* = 250 (pT1pN0), *n* = 131 (pT2pN0), *n* = 310 (pT1-2pN+); TNBC *n* = 638 (pT1pN0), *n* = 413 (pT2pN0), *n* = 452 (pT1-2pN+). Logrank tests of survival differences by grade: ER+PR+HER2− *p* < 0.001, ER+PR−HER2− *p* < 0.001, ER+PR+HER2+ *p* = 0.0001, ER+PR−HER2+ *p* = 0.6100, HER2pos *p* = 0.0099, TNBC *p* < 0.001. Hazard rate curves only plotted until the last event in each group. Hazard ratios adjusted for age and year at diagnosis, subtype × pTN interaction, grade, surgery type and follow-up. *N* = 16,809. Estimates of HRs are given in Additional file 9

Breast cancer survival and early and late mortality rates by IHC subtype and pTN status are presented in Fig. 2. Breast cancer survival was significantly worse with increasing tumour size and with nodal spread within all IHC subtypes, with the possible exception of ER+PR−HER2+ (Fig. 2a–f). Among ER+ subtypes, larger size and nodal spread were associated with increased mortality throughout 13 years of follow-up (Fig. 2g–j). In the adjusted analysis, in particular for ER+PR+HER2− and ER+PR−HER2− subtypes, both larger size (pT2pN0) and nodal spread (pT1-2pN+) were associated with increased early and late mortality (Fig. 2m, n). Among HER2-positive and TNBC subtypes, size and nodal spread were mainly associated with early mortality (TNBC pT1-2pN+ early: HR = 12.9 (8.8–18.9), late: HR = 1.6 (0.8–3.0)) (Fig. 2r). Again, it is important to recall that the mortality in the comparison group (ER+PR+HER2− pT1pN0) increased over follow-up (Fig. 2g).

### Sensitivity analyses

Adjustment for adjuvant treatment in a subset of patients with available treatment information indicated that the associations were essentially unchanged after adjustment for adjuvant treatment, although due to sparser data the models were simplified (Additional file 10). For comparison to other studies, we estimated hazard ratios of IHC subtype without stratification by grade, but with adjustment for grade (Additional file 11). To account for age differences in the prevalence of IHC subtypes, we also present age-standardized Kaplan-Meier curves (Additional files 12 and 13) indicating that age confounding was small in Figs. 1 and 2.

## Discussion

Our study is the first registry-based study of breast cancer death that combines IHC subtype with grade, tumour size and nodal status into high-resolution detailed patient strata. A main finding was that IHC subtype, grade and pTN status were independent prognostic factors for breast cancer death. The largest population-based study ever to assess breast cancer death by IHC subtype utilized SEER registry data [16]. However, the authors did not separate groups by grade or N status, nor did they separate the ER+PR+HER2− group from the smaller ER+PR−HER2− group. A thorough study from the California Cancer Registry [17] stratified survival by IHC markers and AJCC stage, yet was restricted to 5 years of follow-up and only separated the ER+HER2− group by grade.

Of particular importance was the finding that when combining all parameters (and thus defining 48 subgroups of patients), a huge diversity in prognosis was found between the patient groups with a 20- to 40-fold higher rate of breast cancer death in the groups of worst prognosis compared to the group with best prognosis. Further, the consistent finding of a poorer prognosis with increasing tumour size within all levels of IHC subtype and grade, also among low-grade ER+HER2− tumours, suggests that late diagnosis will compromise survival regardless of IHC subtype and grade. Still, the finding that among small tumours (≤ 20 mm) with no nodal spread, ER+PR+HER2− subtype of grade III was associated with the same high mortality as TNBC grade III tumours strengthens the importance of subgroup definition also in small tumours.

Breast cancer patients can suffer relapse and death due to their disease even decades after diagnosis. When analysing the subgroups with regard to timing of death, we found that high grade and nodal spread represented the strongest predictors of late breast cancer death (> 5 years of diagnosis) in ER+HER2− subtypes, and less so in ER+HER2+ subtypes. Contrary, among women with ER− subtypes, the breast cancer mortality was substantially higher close to diagnosis (< 5 years) and of similar magnitude for intermediate- and high-grade tumours. Among ER+ subtypes, there was an indication that PR− status may be associated with worse early mortality (< 5 years), and PR− status consistently conferred a higher grade and Ki67 expression. Adverse effects of PR− on mortality among ER+ subtypes have also been reported in smaller samples [32], but the difference in early vs. late mortality for this group has not been described before.

It has been suggested that low- and high-grade ER+ tumours constitute two independent pathobiological entities with their own characteristics and that intermediate grade is a poorly classified mix of those two underlying types [33–36]. Molecular subtyping complemented with DNA copy number analysis (CNA) suggests that up to eleven subtypes can be identified [11, 37]. Some of these molecular subgroups (within ER+HER2− patients) show an increased risk for late relapse [38]. We found that ER+HER2− tumours of intermediate grade have a survival in-between those of low and high grade, while for ER+HER2+ tumours there was no difference in survival between intermediate and high grade. Breast cancer is recognized as several biologically and clinically distinct subtypes, and in particular ER+ breast cancer is considered to be a spectrum of diseases by international guidelines [5, 7]. According to the ASCO guidelines, the ER+ HER2− subgroup needs stratification into low, intermediate and high risk of relapse to guide adjuvant treatment decisions [39, 40]. Many countries, including Norway, have used the proliferation marker Ki67 for this purpose. However, in our study, the differences in Ki67 level across IHC subtypes were mostly explained by grade. Importantly, among the clinically challenging ER+HER2− intermediate-grade tumours, Ki67 expression did not discriminate between the tumours. It is known internationally that Ki67 measurements display a large variation both with regard to semi-quantitative estimation and cutoff level across laboratories [41, 42]. As some previous studies have questioned the prognostic value of PR status [43], it is of particular interest that our combined analysis shows that subsets of patients with ER+PR−HER2− status have a significantly worse prognosis compared to the ER+PR+HER2− patients. Similar findings of the possible importance of PR were observed in the Californian study [17] and in a Swedish study [44].

Our findings highlight the importance of molecular testing in specific subgroups and the need to integrate molecular subtype with pTN status to predict late risk of recurrence and death. One recommendation for the ER+HER2− group is to implement multimarker molecular-based risk scores [45–47]. These new multigene signatures are expensive and will only be beneficial to implement for subsets of patients. Thus, there will also in the future be a need for evaluating clinically available tumour markers in large patient datasets and monitoring their impact on patient survival. Such analyses can be used as a benchmark for future molecular studies by representing all patients in the population with sufficient numbers in subgroups. This may also help molecular scientists identify which subgroups of patients should be sampled for genetic studies.

It is not surprising that adjustment for treatment did not change the findings of our study, since IHC subtype, grade and pTN status determine the treatment choice. Endocrine treatment, trastuzumab and chemotherapy were routinely used in Norway according to treatment guidelines during the study period [26].

This is one of the largest population-based studies to date evaluating the combined effect of IHC subtype, histological grade, Ki67, tumour size and nodal spread on breast cancer death. The nationwide cancer registry data ensured essentially complete case ascertainment and follow-up for death and migration via routine population registers including all patients presenting at the clinics [22]. The information on subtype was prospectively collected and coded at the CRN throughout the study period [20]. Norway has national treatment guidelines for breast cancer applied at all cancer hospitals [25]. Using thorough adjustments and appropriate modelling of time enabled precise estimation of effects over follow-up. Grade-mix in the reference group of ER+HER2− may be a problem in previous population-based analyses comparing IHC subtypes without adjustment for grade, which would lead to under-estimation of associations. Our supplemental results further highlight the importance of stratifying IHC subtype (ER/PR/HER2) by grade rather than adjusting for grade.

Despite a large nationwide cohort of recently diagnosed patients, the assessment of some combinations of tumour characteristics was not possible due to small numbers. In particular, we could not include Ki67 in the survival analysis. It should be noted that estimates for early (0–5 years) and late (5–13 years) follow-up represent average effects in those time windows and that the window 5–13 years, in particular, include patients with differential follow-up since only a fraction of patients were followed for a full 13 years. No adjustment for socioeconomic status was possible in our dataset; however, the tax-funded healthcare system in Norway is characterized by equal access to diagnostics and treatment across the population, in addition to a national screening programme for women aged 50–69; thus, socioeconomic differences are unlikely to substantially influence the observed associations. We did not have information to adjust for screening; however, age-standardized results indicated no or small influence of age (as proxy for screening) on overall findings though the majority of cases were post-menopausal.

## Conclusion

These results show that tumour size and nodal status, as well as IHC subtype and grade, are important independent predictors of breast cancer death also in patients under modern treatment regimes and therefore must be assessed jointly for their impact on prognosis. In addition, these population-based findings highlight that also patients with ER+ node-negative tumours of low or intermediate grade are in need of new multigene molecular signatures for better prognostic stratification. These findings show the importance of high-quality registry data for evaluating the clinical impact of new multigene signatures, which will be particularly important in the next decades as many countries now include multigene molecular analysis for treatment decisions.

## Supplementary Information

**Additional file 1:** **Figure S1**. Included patients in the analyses.

**Additional file 2:** **Figure S2**. Definition of IHC subtype by grade.

**Additional file 3:** **Figure S3**. Distributions of Ki67 by grade for women diagnosed 2011–2015.

**Additional file 4:** **Figure S4**. Distributions of grade and Ki67 by IHC subtype in women diagnosed 2011–2015.

**Additional file 5:** **Figure S5**. Box plot of Ki67 by grade in women diagnosed 2011–2015.

**Additional file 6:** **Table S6**. Chi-square tests of clinicopathological characteristics by IHC subtype.

**Additional file 7:** **Figure S7**. Distributions of pTN status by IHC subtype and grade. Restricted to M0.

**Additional file 8:** **Figure S8**. Distributions of TNM stage by IHC subtype and grade.

**Additional file 9:** **Table S9**. Adjusted hazard ratios (HR) of BC death for early (0-5y) and late (5-13y) follow-up.

**Additional file 10:** **Table S10**. Adjusted hazard ratios (HR) of BC death with and without adjustment for adjuvant therapy, in subset of patients with adjuvant treatment available.

**Additional file 11: Table S11**. Adjusted hazard ratios (HR) of BC death by IHC subtype with adjustment (rather than stratification) for grade.

**Additional file 12:** **Figure S12**. Kaplan-Meier curves by IHC subtype and grade with and without age-standardisation**.**

**Additional file 13:** **Figure S13**. Kaplan-Meier curves by IHC subtype and pTN status with and without age-standardisation. Restricted to M0.

## Data Availability

The dataset analysed in the current study is not publicly available, but was obtained from the Cancer Registry of Norway under a specific ethical approval by the Regional Committee for Medical and Health Research Ethics in the South East Health Region of Norway. Researchers with appropriate approvals can apply for Norwegian health registry data from https://helsedata.no/.

## Abbreviations

IHC: Immunohistochemistry
ER: Estrogen receptor
PR: Progesterone receptor
HER2: Human epidermal growth factor receptor 2
HER2pos: HER2-positive breast cancer (ER−PR−HER2+)
TNBC: Triple-negative breast cancer (ER−PR−HER2−)
T: Tumour size
N: Nodal status
M: Distant metastasis
CRN: Cancer Registry of Norway
ICD: International Classification of Diseases
pTN: Pathologic T and N status
SEER: Surveillance Epidemiology and End Results programme
CT: Chemotherapy
RT: Radiotherapy
ET: Endocrine therapy
FPM: Flexible parametric survival model
HR: Hazard ratio
CI: Confidence interval
Q1: Quartile 1
Q3: Quartile 3
CNA: Copy number analysis
ASCO: American Society of Clinical Oncology
