# Supplementary material for: In modern times, how important are breast cancer stage, grade and receptor subtype for survival: a population-based cohort study
Source: Breast Cancer Res. 2021 Feb 1;23:17. doi: 10.1186/s13058-021-01393-z (PMC7852363; doi:10.1186/s13058-021-01393-z)
Supplement: Supplementary file 1 — Additional file 1: Figure S1. Included patients in the analyses. [file 13058_2021_1393_MOESM1_ESM.docx]

**Figure S1.** Included patients in the analyses

Women with invasive BC 2005-2015, age 20-74y

N=24,386

Analysed cohort after exclusions (Table 1)

N=24,137

Excluded:

- Not morphologically verified, n=35
- Not confirmed as primary tumours, n=36
- Non-epithelial tumours or Paget’s disease, n=154
- Unclear residency at diagnosis, n=15
- Anaplastic carcinoma, n=9

Analysis of IHC subtype and grade* (Figure 1)

**Restricted to**:

- ER+PR+/-HER2- (grade I,II,III)
- ER+PR+/-HER2+ (grade II, III)
- ER-PR-HER2+/- (grade II, III)

**N=19,220**

Analysis of IHC subtype and pTN status* (Figure 2)

**Restricted to pT1-2pN0/+M0** and:

- ER+PR+/-HER2- (grade I,II,III)
- ER+PR+/-HER2+ (grade II, III)
- ER-PR-HER2+/- (grade II, III)

**N=16,809**

Analysis of IHC subtype, grade and pTN status* (Table 2)

**Restricted to M0** and:

- ER+PR+/-HER2- (grade I,II,III)
- ER+PR+/-HER2+ (grade II, III)
- ER-PR-HER2+/- (grade II, III)

**N=17,204**

* Each analysis only include observations with complete information on all covariates.
