# Supplementary material for: In modern times, how important are breast cancer stage, grade and receptor subtype for survival: a population-based cohort study
Source: Breast Cancer Res. 2021 Feb 1;23:17. doi: 10.1186/s13058-021-01393-z (PMC7852363; doi:10.1186/s13058-021-01393-z)
Supplement: Supplementary file 2 — Additional file 2: Figure S2. Definition of IHC subtype by grade. [file 13058_2021_1393_MOESM2_ESM.docx]

**Figure S2.** Definition of IHC subtype by grade.

ER+

HER2-

HER2+

PR+

PR-

PR-

PR+

ER-

HER2+

HER2-

Set to missing

PR+

PR-

PR-

PR+

**HER2pos**

Set to missing

**TNBC**

N=3036

n=2713

d=250

N=1424

n=1274

d= 99

N=1103

n=939

d=117

N=2114

n=1832

d=314

N=89

N=176

N=13010

n=11891

d=628

Grade II n=6605

d=335

GradeIII

n=1789

d=224

Grade I

n=3497

d=69

N=834

n=721

d=78

ER+PR+

HER2-

ER+PR-

HER2-

ER+PR+

HER2+

ER+PR-

HER2+

Grade II n=1399

d=131

GradeIII

n=717

d=105

Grade I

n=597

d=14

Grade II n=637

d=49

GradeIII

n=564

d=47

Grade I

n=73

d=3

Grade II n=318

d=31

GradeIII

n=368

d=46

Grade I

n=35

d=1

Grade II n=235

d=23

GradeIII

n=689

d=93

Grade I

n=15

d=1

Grade II n=320

d=54

GradeIII

n=1485

d=258

Grade I

n=27

d=2

N = number patients in cohort with known ER/PR/HER2, excluding 2351 women with missing. N=21,786

n = number patients with complete information on ER/PR/HER2, grade, TNM stage, surgery. n=19,220

d = number deaths with complete information on ER/PR/HER2, grade, TNM stage, surgery. n=19,220

Dark grey cells are not included in survival analysis due to few numbers of deaths.
