# Supplementary material for: In modern times, how important are breast cancer stage, grade and receptor subtype for survival: a population-based cohort study
Source: Breast Cancer Res. 2021 Feb 1;23:17. doi: 10.1186/s13058-021-01393-z (PMC7852363; doi:10.1186/s13058-021-01393-z)
Supplement: Supplementary file 3 — Additional file 3: Figure S3. Distributions of Ki67 by grade for women diagnosed 2011–2015. [file 13058_2021_1393_MOESM3_ESM.docx]

**Figure S3.** Distributions of Ki67 by grade for women diagnosed 2011-2015.
