# Supplementary material for: In modern times, how important are breast cancer stage, grade and receptor subtype for survival: a population-based cohort study
Source: Breast Cancer Res. 2021 Feb 1;23:17. doi: 10.1186/s13058-021-01393-z (PMC7852363; doi:10.1186/s13058-021-01393-z)
Supplement: Supplementary file 4 — Additional file 4: Figure S4. Distributions of grade and Ki67 by IHC subtype in women diagnosed 2011–2015. [file 13058_2021_1393_MOESM4_ESM.docx]

**Figure S4.** Distributions of grade and Ki67 by IHC subtype in patients diagnosed 2011-2015.

Based on data 2011-2015 (all panels).

Not available (N/A) if <10 observations.

P-values from Pearson Chi Square test of similar distributions across IHC subtypes (within each panel).

**COMMENT ON RESULTS:**

ER+HER2– tumours were mostly grade I and II, and of varying Ki67, while HER2 positive and TNBC were mainly grade III (75% and 84%, respectively) and high Ki67 expression (Figure 1). ER+HER2+ tumours were in between. More than 96% of grade I tumours were ER+HER2–, and PR– was associated with higher grade and Ki67 compared to PR+ among ER+ tumours (p<0.01, Supplementary Table 1).

Stratifying on grade, Ki67 did not provide discriminatory information for ER+HER2– subtypes of grade II, while among ER+HER2+ subtypes medium and high Ki67 was significantly more common and similar to ER– tumours. Although rare, ER+PR–HER2+ tumours of grade I had significantly higher Ki67 expression levels (p<0.001) compared to ER+PR+HER2+ of grade I. Among grade III tumours, high Ki67 was present among all subtypes, but somewhat less among ER+ tumours.
