# Supplementary material for: In modern times, how important are breast cancer stage, grade and receptor subtype for survival: a population-based cohort study
Source: Breast Cancer Res. 2021 Feb 1;23:17. doi: 10.1186/s13058-021-01393-z (PMC7852363; doi:10.1186/s13058-021-01393-z)
Supplement: Supplementary file 6 — Additional file 6: Table S6. Chi-square tests of clinicopathological characteristics by IHC subtype. [file 13058_2021_1393_MOESM6_ESM.docx]

**Table S6.** Chi-square tests of clinicopathological characteristics by IHC subtype.

| **IHC subtype** | **ER+PR+HER2-** | | **ER+PR-HER2-** | | **ER+PR+HER2+** | | **ER+PR-HER2+** | | **ER-PR-HER2+**  **(HER2pos)** | | **ER-PR-HER2-**  **(TNBC)** | |
| --- | --- | --- | --- | --- | --- | --- | --- | --- | --- | --- | --- | --- |
|  | **N** | **%** | **N** | **%** | **N** | **%** | **N** | **%** | **N** | **%** | **N** | **%** |
| Total | 13010 | 100 | 3036 | 100 | 1424 | 100 | 834 | 100 | 1103 | 100 | 2114 | 100 |
| **Grade** |  |  |  |  |  |  |  |  |  |  |  |  |
| I: Low | 3608 | 29.3 | 614 | 21.7 | 79 | 5.9 | 36 | 4.8 | 15 | 1.5 | 30 | 1.6 |
| II: Medium | 6849 | 55.6 | 1459 | 51.6 | 666 | 50.2 | 331 | 43.8 | 244 | 25.0 | 340 | 17.5 |
| III: High | 1860 | 15.1 | 752 | 26.6 | 583 | 43.9 | 388 | 51.4 | 719 | 73.5 | 1571 | 80.9 |
| Overall test comparing subtypes P<0.001 | Test PR+ vs PR-  P<0.001 | | | | Test PR+ vs PR-  P=0.004 | | | | Test HER2+ vs HER2-  P<0.001 | | | |
| **Ki67^a^** |  |  |  |  |  |  |  |  |  |  |  |  |
| 0-14.9% | 2173 | 37.2 | 388 | 30.5 | 75 | 14.3 | 22 | 6.4 | 15 | 4.3 | 37 | 4.7 |
| 15.0-30.0% | 2138 | 36.6 | 384 | 30.2 | 128 | 24.3 | 106 | 30.9 | 68 | 19.7 | 64 | 8.1 |
| 30.1-100% | 1531 | 26.2 | 501 | 39.4 | 323 | 61.4 | 215 | 62.7 | 263 | 76.0 | 692 | 87.3 |
| Overall test comparing subtypes P<0.001 | Test PR+ vs PR-  P<0.001 | | | | Test PR+ vs PR-  P=0.001 | | | | Test HER2+ vs HER2-  P<0.001 | | | |
| **pT** |  |  |  |  |  |  |  |  |  |  |  |  |
| pT1: 0-20 mm | 8407 | 72.2 | 1735 | 66.9 | 718 | 62.3 | 372 | 58.5 | 434 | 53.5 | 929 | 54.6 |
| pT2: 21-50 mm | 2932 | 25.2 | 778 | 30.0 | 400 | 34.7 | 242 | 38.1 | 329 | 40.6 | 725 | 42.6 |
| pT3: >50 mm | 186 | 1.6 | 55 | 2.1 | 19 | 1.6 | 12 | 1.9 | 22 | 2.7 | 23 | 1.4 |
| pT4: spread, any size | 115 | 1.0 | 24 | 0.9 | 16 | 1.4 | 10 | 1.6 | 26 | 3.2 | 23 | 1.4 |
| Overall test comparing subtypes P<0.001 | Test PR+ vs PR-  P<0.001 | | | | Test PR+ vs PR-  P=0.479 | | | | Test HER2+ vs HER2-  P<0.001 | | | |
| **pN** |  |  |  |  |  |  |  |  |  |  |  |  |
| pN0: 0 nodes+ | 8141 | 67.4 | 1816 | 65.2 | 716 | 55.3 | 376 | 50.9 | 441 | 45.3 | 1196 | 63.1 |
| pN1: 1-3 nodes+ | 3333 | 27.6 | 819 | 29.4 | 479 | 37.0 | 282 | 38.2 | 424 | 43.5 | 599 | 31.6 |
| pN2: 4-9 nodes+ | 413 | 3.4 | 90 | 3.2 | 73 | 5.6 | 55 | 7.4 | 67 | 6.9 | 62 | 3.3 |
| pN3: 10+ nodes+ | 187 | 1.5 | 61 | 2.2 | 27 | 2.1 | 26 | 3.5 | 42 | 4.3 | 39 | 2.1 |
| Overall test comparing subtypes P<0.001 | Test PR+ vs PR-  P=0.018 | | | | Test PR+ vs PR-  P=0.046 | | | | Test HER2+ vs HER2-  P<0.001 | | | |
| **Stage** |  |  |  |  |  |  |  |  |  |  |  |  |
| I | 6619 | 53.0 | 1406 | 48.6 | 519 | 38.2 | 266 | 33.8 | 296 | 28.4 | 730 | 36.8 |
| II | 4519 | 36.2 | 1117 | 38.6 | 589 | 43.3 | 324 | 41.2 | 465 | 44.6 | 953 | 48.1 |
| III | 1056 | 8.5 | 285 | 9.8 | 185 | 13.6 | 144 | 18.3 | 225 | 21.6 | 234 | 11.8 |
| IV | 287 | 2.3 | 88 | 3.0 | 66 | 4.9 | 53 | 6.7 | 56 | 5.4 | 65 | 3.3 |
| Overall test comparing subtypes P<0.001 | Test PR+ vs PR-  P<0.001 | | | | Test PR+ vs PR-  P=0.004 | | | | Test HER2+ vs HER2-  P<0.001 | | | |

^a^ Ki67 only available diagnosis years 2011-2015
