# Supplementary material for: In modern times, how important are breast cancer stage, grade and receptor subtype for survival: a population-based cohort study
Source: Breast Cancer Res. 2021 Feb 1;23:17. doi: 10.1186/s13058-021-01393-z (PMC7852363; doi:10.1186/s13058-021-01393-z)
Supplement: Supplementary file 7 — Additional file 7: Figure S7. Distributions of pTN status by IHC subtype and grade. Restricted to M0. [file 13058_2021_1393_MOESM7_ESM.docx]

**Figure S7.** Distributions of pTN status by IHC subtype and grade. Restricted to M0.

P-values from Pearson Chi Square test of similar distributions across IHC subtypes (within each panel).

**COMMENTS ON RESULTS:**

Among patients with non-metastatic disease, 57% of ER+PR+HER2– and 54% of ER+PR–HER2– tumours were smaller than 20 mm and had no nodal spread (pT1pN0) while the proportions ranged from 35% to 45% for the other subtypes. ER+HER2+ and HER2 positive subtypes had the highest proportions of small tumours with nodal spread (pT1-2pN+) (range 37% to 42%), while TNBC had similar proportion of pT1-2pN+ to ER+HER2–.

Stratifying on grade, the pTN distributions were similar among grade I (p=0.747) and grade II (p=0.113) (Figure 2, panel 1 and 2), while among grade III tumours TNBC subtype had smaller tumours with less nodal involvement compared to the other subtypes (p<0.001).
