# Supplementary material for: In modern times, how important are breast cancer stage, grade and receptor subtype for survival: a population-based cohort study
Source: Breast Cancer Res. 2021 Feb 1;23:17. doi: 10.1186/s13058-021-01393-z (PMC7852363; doi:10.1186/s13058-021-01393-z)
Supplement: Supplementary file 8 — Additional file 8: Figure S8. Distributions of TNM stage by IHC subtype and grade. [file 13058_2021_1393_MOESM8_ESM.docx]

**Figure S8.** Distributions of TNM stage by IHC subtype and grade.

P-values from Pearson Chi Square test of similar distributions across IHC subtypes (within each panel).
