# Supplementary material for: In modern times, how important are breast cancer stage, grade and receptor subtype for survival: a population-based cohort study
Source: Breast Cancer Res. 2021 Feb 1;23:17. doi: 10.1186/s13058-021-01393-z (PMC7852363; doi:10.1186/s13058-021-01393-z)
Supplement: Supplementary file 9 — Additional file 9: Table S9. Adjusted hazard ratios (HR) of BC death for early (0-5y) and late (5-13y) follow-up. [file 13058_2021_1393_MOESM9_ESM.docx]

**Table S9.** Adjusted hazard ratios (HR) of BC death for early (0-5y) and late (5-13y) follow-up.

|  |  | **Patients**  **/Deaths** | **Patients**  **/Deaths** | **Model (1)** | |
| --- | --- | --- | --- | --- | --- |
|  |  | **0-5y** | **5-13y** | **0-5y** | **5-13y** |
| **IHC subtype** | **Grade** | **N/n** | **N/n** | **HR [95% CI]** | **HR [95% CI]** |
| ER+ PR+ HER2- | I | 3497/30 | 2241/39 | 1.0 [ref] | 1.0 [ref] |
|  | II | 6605/176 | 4090/159 | 1.4 [1.0,1.9] | 1.8 [1.3,2.5] |
|  | III | 1789/155 | 996/69 | 3.9 [2.9,5.3] | 2.9 [2.0,4.1] |
|  |  |  |  |  |  |
| ER+ PR- HER2- | I | 597/8 | 370/6 | 1.2 [0.6,2.6] | 1.2 [0.5,2.9] |
|  | II | 1399/89 | 858/42 | 3.2 [2.3,4.5] | 2.1 [1.4,3.2] |
|  | III | 717/76 | 373/29 | 5.6 [3.9,7.9] | 3.9 [2.4,6.1] |
|  |  |  |  |  |  |
| ER+ PR+ HER2+ | II | 637/28 | 408/21 | 1.5 [0.9,2.3] | 1.9 [1.2,3.2] |
|  | III | 564/24 | 363/23 | 1.6 [1.0,2.7] | 2.5 [1.6,4.2] |
|  |  |  |  |  |  |
| ER+ PR- HER2+ | II | 318/21 | 201/10 | 2.0 [1.2,3.4] | 1.7 [0.9,3.4] |
|  | III | 368/33 | 199/13 | 3.3 [2.2,5.1] | 2.6 [1.4,4.7] |
|  |  |  |  |  |  |
| HER2pos | II | 235/18 | 143/5 | 2.7 [1.6,4.5] | 1.2 [0.5,3.1] |
|  | III | 689/77 | 412/16 | 3.7 [2.6,5.3] | 1.2 [0.7,2.2] |
|  |  |  |  |  |  |
| TNBC | II | 320/40 | 195/14 | 7.8 [5.2,11.7] | 3.9 [2.2,7.0] |
|  | III | 1485/233 | 821/25 | 10.3 [7.6,13.8] | 1.6 [1.0,2.5] |
|  |  |  |  |  |  |
|  |  | **Patients**  **/Deaths** | **Patients**  **/Deaths** | **Model (2)** | |
|  |  | **0-5y** | **5-13y** | **0-5y** | **5-13y** |
| **IHC subtype** | **pTpN** | **N/n** | **N/n** | **HR [95% CI]** | **HR [95% CI]** |
| ER+ PR+ HER2- | pT1 pN0 | 6229/29 | 3868/49 | 1.0 [ref] | 1.0 [ref] |
|  | pT2 pN0 | 1362/31 | 819/23 | 2.7 [1.7,4.3] | 1.9 [1.2,3.2] |
|  | pT1-2 pN+ | 3125/92 | 2027/130 | 3.5 [2.4,4.9] | 4.4 [3.2,6.0] |
|  |  |  |  |  |  |
| ER+ PR- HER2- | pT1 pN0 | 1309/17 | 799/12 | 2.0 [1.2,3.5] | 1.4 [0.7,2.5] |
|  | pT2 pN0 | 361/17 | 186/10 | 4.9 [2.8,8.7] | 3.3 [1.7,6.5] |
|  | pT1-2 pN+ | 694/64 | 441/37 | 9.1 [6.2,13.3] | 4.8 [3.1,7.4] |
|  |  |  |  |  |  |
| ER+ PR+ HER2+ | pT1 pN0 | 437/3 | 288/2 | 0.7 [0.2,2.2] | 0.4 [0.1,1.6] |
|  | pT2 pN0 | 171/4 | 106/6 | 2.1 [0.8,5.9] | 3.5 [1.5,8.1] |
|  | pT1-2 pN+ | 380/14 | 267/15 | 2.9 [1.6,5.2] | 2.6 [1.5,4.7] |
|  |  |  |  |  |  |
| ER+ PR- HER2+ | pT1 pN0 | 225/7 | 135/6 | 3.1 [1.4,6.9] | 2.6 [1.1,6.1] |
|  | pT2 pN0 | 92/3 | 55/4 | 2.6 [0.8,8.5] | 4.2 [1.5,11.6] |
|  | pT1-2 pN+ | 230/15 | 141/3 | 5.5 [3.0,9.9] | 1.1 [0.3,3.6] |
|  |  |  |  |  |  |
| HER2pos | pT1 pN0 | 250/7 | 168/4 | 2.2 [1.0,4.9] | 0.9 [0.3,2.5] |
|  | pT2 pN0 | 131/8 | 75/1 | 4.4 [2.1,9.4] | 0.6 [0.1,4.0] |
|  | pT1-2 pN+ | 310/26 | 202/9 | 5.7 [3.5,9.4] | 1.7 [0.8,3.6] |
|  |  |  |  |  |  |
| TNBC | pT1 pN0 | 638/24 | 413/16 | 3.0 [1.8,5.0] | 1.7 [1.0,3.0] |
|  | pT2 pN0 | 413/42 | 238/5 | 7.4 [4.8,11.5] | 0.8 [0.3,2.1] |
|  | pT1-2 pN+ | 452/81 | 260/11 | 12.9 [8.8,18.9] | 1.6 [0.8,3.0] |
|  |  |  |  |  |  |

**HER2pos**=ER-PR-HER2+; **TNBC**=ER-PR-HER2-

Model (1) adjusted for subtype x grade interaction, age and year at diagnosis, follow-up, TNM stage and surgery type. N=19220.

Model (2) adjusted for subtype x pTN status interaction, age and year at diagnosis, follow-up, grade and surgery type. N=16809. Restricted to M0.
