# Supplementary material for: In modern times, how important are breast cancer stage, grade and receptor subtype for survival: a population-based cohort study
Source: Breast Cancer Res. 2021 Feb 1;23:17. doi: 10.1186/s13058-021-01393-z (PMC7852363; doi:10.1186/s13058-021-01393-z)
Supplement: Supplementary file 10 — Additional file 10: Table S10. Adjusted hazard ratios (HR) of BC death with and without adjustment for adjuvant therapy, in subset of patients with adjuvant treatment available. [file 13058_2021_1393_MOESM10_ESM.docx]

**Table S10.** Adjusted hazard ratios (HR) of BC death with and without adjustment for adjuvant therapy, in subset of patients with adjuvant treatment available.

|  |  | **Full data**  **N=19,220** | **Data with adjuvant treatment**  **N=10,990** | |
| --- | --- | --- | --- | --- |
|  |  | **Model (1)**  Adjusted for surgery | **Model (2)**  Adjusted for surgery | **Model (3)**  Adjusted for, surgery and adjuvant therapy |
| **IHC subtype** | **Grade** | **HR [95% CI]** | **HR [95% CI]** | **HR [95% CI]** |
| ER+ PR+ HER2- | I | 1.0 [ref] | 1.0 [ref] | 1.0 [ref] |
|  | II | 1.6 [1.2,2.0] | 1.5 [1.1,2.1] | 1.5 [1.1,2.1] |
|  | III | 3.4 [2.6,4.5] | 3.2 [2.3,4.6] | 3.2 [2.2,4.5] |
|  |  |  |  |  |
| ER+ PR- HER2- | I | 1.2 [0.7,2.2] | 1.4 [0.7,2.7] | 1.4 [0.7,2.7] |
|  | II | 2.7 [2.0,3.6] | 2.4 [1.7,3.6] | 2.4 [1.6,3.4] |
|  | III | 4.8 [3.5,6.5] | 4.1 [2.8,6.2] | 4.1 [2.7,6.0] |
|  |  |  |  |  |
| ER+ PR+ HER2+ | II | 1.6 [1.1,2.3] | 1.5 [0.9,2.5] | 1.5 [0.9,2.4] |
|  | III | 1.9 [1.3,2.8] | 1.7 [1.1,2.7] | 1.7 [1.1,2.7] |
|  |  |  |  |  |
| ER+ PR- HER2+ | II | 1.9 [1.2,2.9] | 1.4 [0.8,2.6] | 1.4 [0.8,2.6] |
|  | III | 2.9 [2.0,4.3] | 2.1 [1.3,3.5] | 2.1 [1.3,3.5] |
|  |  |  |  |  |
| HER2pos | II | 2.1 [1.3,3.3] | 1.5 [0.7,3.0] | 1.6 [0.8,3.3] |
|  | III | 2.7 [2.0,3.7] | 2.5 [1.6,3.8] | 2.7 [1.7,4.2] |
|  |  |  |  |  |
| TNBC | II | 6.1 [4.2,8.7] | 3.9 [2.3,6.5] | 4.4 [2.6,7.4] |
|  | III | 6.5 [5.0,8.6] | 5.7 [4.0,8.2] | 6.4 [4.4,9.3] |
|  |  |  |  |  |
|  |  | **Full data**  **N=16,809** | **Data with adjuvant treatment**  **N=9,761** | |
|  |  | **Model (4)**  Adjusted for surgery | **Model (5)**  Adjusted for surgery | **Model (6)**  Adjusted for, surgery and adjuvant therapy |
| **IHC subtype** | **pTN** | **HR [95% CI]** | **HR [95% CI]** | **HR [95% CI]** |
| ER+ PR+ HER2- | pT1 pN0 | 1.0 [ref] | 1.0 [ref] | 1.0 [ref] |
|  | pT2 pN0 | 2.3 [1.6,3.3] | 2.2 [1.4,3.3] | 2.2 [1.4,3.4] |
|  | pT1-2 pN+ | 3.9 [3.0,5.1] | 3.6 [2.7,4.9] | 3.3 [2.4,4.6] |
|  |  |  |  |  |
| ER+ PR- HER2- | pT1 pN0 | 1.7 [1.1,2.6] | 1.4 [0.8,2.4] | 1.4 [0.8,2.3] |
|  | pT2 pN0 | 4.1 [2.6,6.3] | 3.7 [2.1,6.4] | 3.7 [2.1,6.5] |
|  | pT1-2 pN+ | 6.8 [5.0,9.3] | 6.4 [4.4,9.2] | 5.8 [3.9,8.5] |
|  |  |  |  |  |
| ER+ PR+ HER2+ | pT1 pN0 | 0.5 [0.2,1.3] | 0.5 [0.2,1.4] | 0.5 [0.2,1.4] |
|  | pT2 pN0 | 2.7 [1.4,5.3] | 2.4 [1.1,5.4] | 2.4 [1.1,5.3] |
|  | pT1-2 pN+ | 2.8 [1.8,4.3] | 1.9 [1.1,3.2] | 1.7 [1.0,3.0] |
|  |  |  |  |  |
| ER+ PR- HER2+ | pT1 pN0 | 2.8 [1.6,5.1] | 2.4 [1.2,4.9] | 2.4 [1.2,4.9] |
|  | pT2 pN0 | 3.2 [1.5,7.0] | 2.0 [0.7,5.7] | 2.0 [0.7,5.6] |
|  | pT1-2 pN+ | 3.3 [1.9,5.5] | 1.4 [0.6,3.3] | 1.3 [0.5,3.1] |
|  |  |  |  |  |
| HER2pos | pT1 pN0 | 1.5 [0.8,2.8] | 1.3 [0.5,3.0] | 1.4 [0.6,3.2] |
|  | pT2 pN0 | 2.4 [1.2,4.9] | 0.8 [0.2,3.2] | 0.8 [0.2,3.5] |
|  | pT1-2 pN+ | 3.6 [2.4,5.5] | 1.8 [0.9,3.6] | 1.9 [1.0,3.7] |
|  |  |  |  |  |
| TNBC | pT1 pN0 | 2.3 [1.6,3.5] | 2.2 [1.3,3.7] | 2.4 [1.4,4.0] |
|  | pT2 pN0 | 4.0 [2.7,5.9] | 2.8 [1.6,4.8] | 3.1 [1.7,5.6] |
|  | pT1-2 pN+ | 6.9 [5.0,9.6] | 5.8 [3.8,8.8] | 6.2 [3.9,10.0] |
|  |  |  |  |  |

**HER2pos**=ER-PR-HER2+; **TNBC**=ER-PR-HER2-

Model (1) adjusted for: subtype, grade, age and year of diagnosis, follow-up, TNM stage and surgery.

Model (2) adjusted for: subtype, grade, age and year of diagnosis, follow-up, TNM stage and surgery.

Model (3) adjusted for: subtype, grade, age and year of diagnosis, follow-up, TNM stage, surgery and adjuvant treatment (RT, CT, ET).

Model (4) adjusted for: IHC subtype, pTN, age and year of diagnosis, follow-up, grade and surgery.

Model (5) adjusted for: IHC subtype, pTN, age and year of diagnosis, follow-up, grade and surgery.

Model (6) adjusted for: IHC subtype, pTN, age and year of diagnosis, follow-up, grade, surgery and adjuvant treatment (RT, CT, ET).

Model (4)-(6) on patients restricted to pT1-2 pN0/+ M0.
