# Supplementary material for: In modern times, how important are breast cancer stage, grade and receptor subtype for survival: a population-based cohort study
Source: Breast Cancer Res. 2021 Feb 1;23:17. doi: 10.1186/s13058-021-01393-z (PMC7852363; doi:10.1186/s13058-021-01393-z)
Supplement: Supplementary file 11 — Additional file 11: Table S11. Adjusted hazard ratios (HR) of BC death by IHC subtype with adjustment (rather than stratification) for grade. [file 13058_2021_1393_MOESM11_ESM.docx]

**Table S11.** Adjusted hazard ratios (HR) of BC death by IHC subtype with adjustment (rather than stratification) for grade.

|  |  | **Patients**  **/Deaths** | **Patients**  **/Deaths** | **Model (1)**  Adjusted age, year,  IHC subtype, grade,  TNM stage and surgery | |
| --- | --- | --- | --- | --- | --- |
|  |  | **0-5 y** | **5-13 y** | **0-5 y** | **5-13 y** |
| **IHC subtype** | **Grade** | **N/n** | **N /n** | **HR [95% CI]** | **HR [95% CI]** |
|  |  |  |  |  |  |
| ER+ PR+ HER2- | I-III | 11891 /361 | 7327 /267 | 1.0 [ref] | 1.0 [ref] |
| ER+ PR- HER2- | I-III | 2713 /173 | 1601 /77 | 1.8 [1.5,2.2] | 1.2 [1.0,1.6] |
| ER+ PR+ HER2+ | II-III | 1201 /52 | 771 /44 | 0.7 [0.5,0.9] | 0.9 [0.7,1.3] |
| ER+ PR- HER2+ | II-III | 686 /54 | 400 /23 | 1.1 [0.9,1.5] | 0.9 [0.6,1.4] |
| HER2pos | II-III | 924 /95 | 555 /21 | 1.3 [1.0,1.6] | 0.4 [0.3,0.7] |
| TNBC | II-III | 1805 /273 | 1016 /39 | 3.6 [3.0,4.2] | 0.7 [0.5,1.0] |
|  |  |  |  |  |  |
| **Total** |  | 19220 /1008 | 11670 /471 |  |  |

**HER2pos**=ER-PR-HER2+; **TNBC**=ER-PR-HER2-

**This analysis is in comparison to the hazard ratios in Figure 1, where IHC subtype is stratified by grade.**

For comparison to previous registry-based studies, we estimated hazard ratios of IHC subtype without stratification by grade, but with adjustment for grade (Supplemental Table S10). In this analysis, the subtype hazard ratios were compared to the total ER+PR+HER2– group assuming the same average effect across all levels of grade. However, since the effect of grade is varying over IHC subtypes, adjustment for grade, rather than stratification, will hide interaction effects between IHC subtype and grade. Because the reference group is an average across grade, the hazard ratios will be lower compared to a stratified analysis, where the reference group is ER+PR+HER2- grade I.

This supplemental result highlights the importance of stratifying IHC subtype (ER/PR/HER2) by grade rather than adjusting for grade in the presence of interactions.
