# Supplementary material for: In modern times, how important are breast cancer stage, grade and receptor subtype for survival: a population-based cohort study
Source: Breast Cancer Res. 2021 Feb 1;23:17. doi: 10.1186/s13058-021-01393-z (PMC7852363; doi:10.1186/s13058-021-01393-z)
Supplement: Supplementary file 12 — Additional file 12: Figure S12. Kaplan-Meier curves by IHC subtype and grade with and without age-standardisation. [file 13058_2021_1393_MOESM12_ESM.docx]

**Figure S12.** Kaplan-Meier curves by IHC subtype and grade with and without age-standardisation**.**

Age-standardised according to overall age-distribution in the sample using age groups (0-44: 14%, 45-54: 29%, 55-64: 33%, 65-74: 24%).
