# Supplementary material for: In modern times, how important are breast cancer stage, grade and receptor subtype for survival: a population-based cohort study
Source: Breast Cancer Res. 2021 Feb 1;23:17. doi: 10.1186/s13058-021-01393-z (PMC7852363; doi:10.1186/s13058-021-01393-z)
Supplement: Supplementary file 13 — Additional file 13: Figure S13. Kaplan-Meier curves by IHC subtype and pTN status with and without age-standardisation. Restricted to M0. [file 13058_2021_1393_MOESM13_ESM.docx]

**Figure S13.** Kaplan-Meier curves by IHC subtype and pTN status with and without age-standardisation. Restricted to M0.

Age-standardised according to overall age-distribution in the sample using age groups (0-44: 14%, 45-54: 29%, 55-64: 33%, 65-74: 24%).
